# Single-molecule RNA detection at depth by hybridization chain reaction and tissue hydrogel embedding and clearing

**DOI:** 10.1242/dev.138560

**Published:** 2016-08-01

**Authors:** Sheel Shah, Eric Lubeck, Maayan Schwarzkopf, Ting-Fang He, Alon Greenbaum, Chang Ho Sohn, Antti Lignell, Harry M. T. Choi, Viviana Gradinaru, Niles A. Pierce, Long Cai

**Affiliations:** 1 Division of Chemistry and Chemical Engineering, California Institute of Technology, Pasadena, CA 91125, USA; 2 UCLA-Caltech Medical Scientist Training Program, David Geffen School of Medicine, University of California at Los Angeles, Los Angeles, CA 90095, USA; 3 Division of Biology and Biological Engineering, California Institute of Technology, Pasadena, CA 91125, USA; 4 Division of Engineering & Applied Science, California Institute of Technology, Pasadena, CA 91125, USA

**Keywords:** CLARITY, Amplification, Single-molecule RNA

## Abstract

Accurate and robust detection of mRNA molecules in thick tissue samples can reveal gene expression patterns in single cells within their native environment. Preserving spatial relationships while accessing the transcriptome of selected cells is a crucial feature for advancing many biological areas – from developmental biology to neuroscience. However, because of the high autofluorescence background of many tissue samples, it is difficult to detect single-molecule fluorescence *in situ* hybridization (smFISH) signals robustly in opaque thick samples. Here, we draw on principles from the emerging discipline of dynamic nucleic acid nanotechnology to develop a robust method for multi-color, multi-RNA imaging in deep tissues using single-molecule hybridization chain reaction (smHCR). Using this approach, single transcripts can be imaged using epifluorescence, confocal or selective plane illumination microscopy (SPIM) depending on the imaging depth required. We show that smHCR has high sensitivity in detecting mRNAs in cell culture and whole-mount zebrafish embryos, and that combined with SPIM and PACT (passive CLARITY technique) tissue hydrogel embedding and clearing, smHCR can detect single mRNAs deep within thick (0.5 mm) brain slices. By simultaneously achieving ∼20-fold signal amplification and diffraction-limited spatial resolution, smHCR offers a robust and versatile approach for detecting single mRNAs *in situ*, including in thick tissues where high background undermines the performance of unamplified smFISH.

## Abstract (precis)

**Summary:** Single-molecule hybridization chain reaction, combined with tissue clearing, allows the near-quantitative and spatially localized detection of mRNAs in thick tissue samples.

## INTRODUCTION

Imaging gene expression levels with single-cell resolution in intact tissues is essential for understanding the genetic programs in many systems, such as developing embryos and dynamic brain circuits. Single-molecule fluorescence *in situ* hybridization (smFISH) has been the standard tool for detection of individual RNAs in cells (Femino et al., 1998; Raj et al., 2008, 2006; Levsky et al., 2002; Fan et al., 2001). Using smFISH, an mRNA is detected by a probe set containing 20-40 DNA probes, each carrying one or more fluorophores, and each complementary to a different short subsequence (20-50 nt) along the mRNA target. This approach ensures that multiple probes bind the mRNA, generating bright puncta that can be discriminated from background staining resulting from non-specific binding of individual probes. However, background due to sample autofluorescence is significantly higher in tissue samples than in cell culture, making it difficult to robustly detect smFISH signals in tissue. In addition, light scattering caused by deep tissue imaging necessitates probes with higher photon counts than for thin section imaging. Although we have shown that tissue clearing by PACT (passive CLARITY technique) can alleviate autofluorescence and light scattering problems (Chung et al., 2013; Treweek et al., 2015; Yang et al., 2014) while preserving RNA molecules (Yang et al., 2014), a more robust signal amplification strategy is needed to enable multi-color mapping of single mRNAs in deep tissues (Fig. S1).

**Fig. 1.**
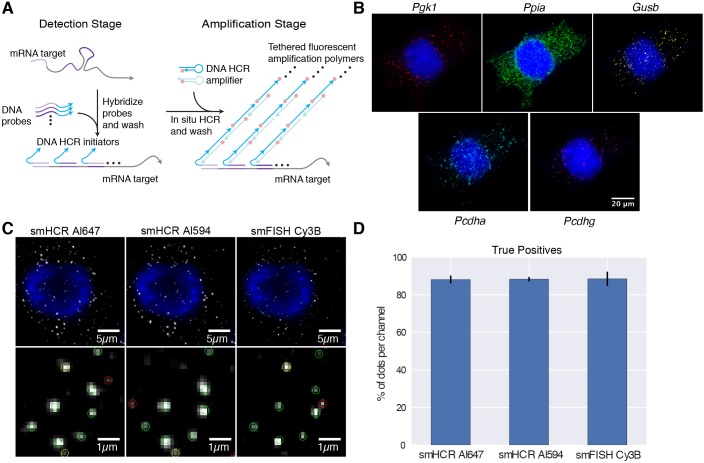
**Single-molecule hybridization chain reaction (smHCR).** (A) smHCR protocol. Detection stage: an mRNA target is detected by a probe set containing 20-40 short DNA probes, each binding a 20-30 nt subsequence of the target; each probe in the probe set carries an initiator for the same HCR amplifier. Amplification stage: metastable fluorophore-labeled DNA HCR hairpins penetrate the sample and self-assemble into fluorescent amplification polymers tethered to their initiating probes. The same two-stage protocol is used for multiplexed studies: during the detection stage, all probe sets are introduced simultaneously, each carrying an initiator for an orthogonal HCR amplifier; during the amplification stage, all HCR amplifiers are introduced simultaneously, each labeled with spectrally distinct fluorophores. (B) Simultaneous mapping of five target mRNAs in cultured CAD cells using five spectrally distinct HCR amplifiers (DAPI in blue): *Pgk1* (Cy7), *Ppia* (Alexa Fluor 647), *Gusb* (Alexa Fluor 594), *Pcdha* (Cy3b), *Pcdhg* (Alexa Fluor 488). (C) Comparison of smHCR and smFISH for detection of *Pgk1* via dot colocalization in three channels: smHCR (Alexa Fluor 647), smHCR (Alexa Fluor 594), smFISH (Cy3B). Dots are classified as triple-detected true positives (present in all three channels; green circles), double-detected true positives (present in two out of three channels; yellow circles), or false positives (present in only one channel; red circles). (D) True positive rates for each channel in C (median±median absolute deviation; *N*=10 wells). Microscopy: epifluorescence. Probe sets for B-D: 22 probes per set, each addressing a 20 nt target subsequence. See Figs S2-S5 for additional data.

Attempts have been made to specifically amplify a single mRNA signal, but these tend to suffer from low efficiency and complex protocols (Player et al., 2001). Here, we describe a simple and efficient method for multiplexed single-molecule signal amplification based on the mechanism of hybridization chain reaction (HCR) (Dirks and Pierce, 2004; Choi et al., 2010, 2014). With this approach, short DNA probes complementary to mRNA targets trigger chain reactions in which metastable fluorophore-labeled DNA hairpins self-assemble into tethered fluorescent amplification polymers (Fig. 1A). As with smFISH, each target mRNA is addressed by 20-40 probes complementary to different subsequences along the target to enable discrimination between mRNAs with multiple probes bound and dots resulting from non-specific binding of individual probes. In contrast to previous *in situ* HCR methods (Choi et al., 2010, 2014), we limit the HCR amplification time to achieve a mean polymer length of ∼20-40 hairpins, generating puncta that are bright enough for high sensitivity, yet small enough for diffraction-limited resolution (Fig. S2). Using orthogonal HCR amplifiers programmed to operate independently, straightforward multiplexing is achieved for up to five channels simultaneously (Fig. 1B, Table S1). We term this method single-molecule HCR (smHCR).

## RESULTS AND DISCUSSION

To characterize the sensitivity and selectivity of smHCR in cultured cells, we performed a colocalization experiment in which a low-copy target mRNA (*Pcdha* constant region) was simultaneously detected using three probe sets of 22 probes each (one smFISH set and two smHCR sets), with the probes alternating between the three sets along the target (Table S2). Dots were identified in each channel by applying a threshold following standard methods for smFISH data analysis (Fig. S3A) (Raj et al., 2008). We define true mRNA signals as those dots that are colocalized in at least two of the three channels (Fig. 1C). We calculate a true positive rate for a given channel as the percentage of true mRNA signals detected as dots in that channel. We calculate a false positive rate for a given channel as the percentage of dots in that channel that are not true mRNA signals. For the two smHCR channels and the smFISH channel, the true positive rates are all approximately 88% (Fig. 1D), and the false positive rates are approximately 36%, 27% and 20%, respectively (Fig. S3B). Comparing dot intensities, smHCR provides signal amplification in the region of ∼15- to 35-fold relative to smFISH (Fig. S3C,D and Fig. S4), a feature that will become crucial in detecting single transcripts in tissues that have higher levels of autofluorescence.

Notably, in experimental designs where two channels can be allocated to each target mRNA, near-quantitative single-molecule mapping can be achieved (Fig. S5). Using this approach, the threshold for dot identification is lowered in each channel to achieve a higher true positive rate (>95%) at the cost of a higher false positive rate (>60%). Dot colocalization between channels can then be used to identify the subset of dots that represent true mRNA signals. Alternatively, for the standard situation where each target is detected in only a single channel, and hence colocalization cannot be used for dot classification, the threshold must be raised to reject false positives at the cost of also rejecting some true positives, as is the case for smFISH (Fig. S5) (Raj et al., 2008).

**Fig. 2.**
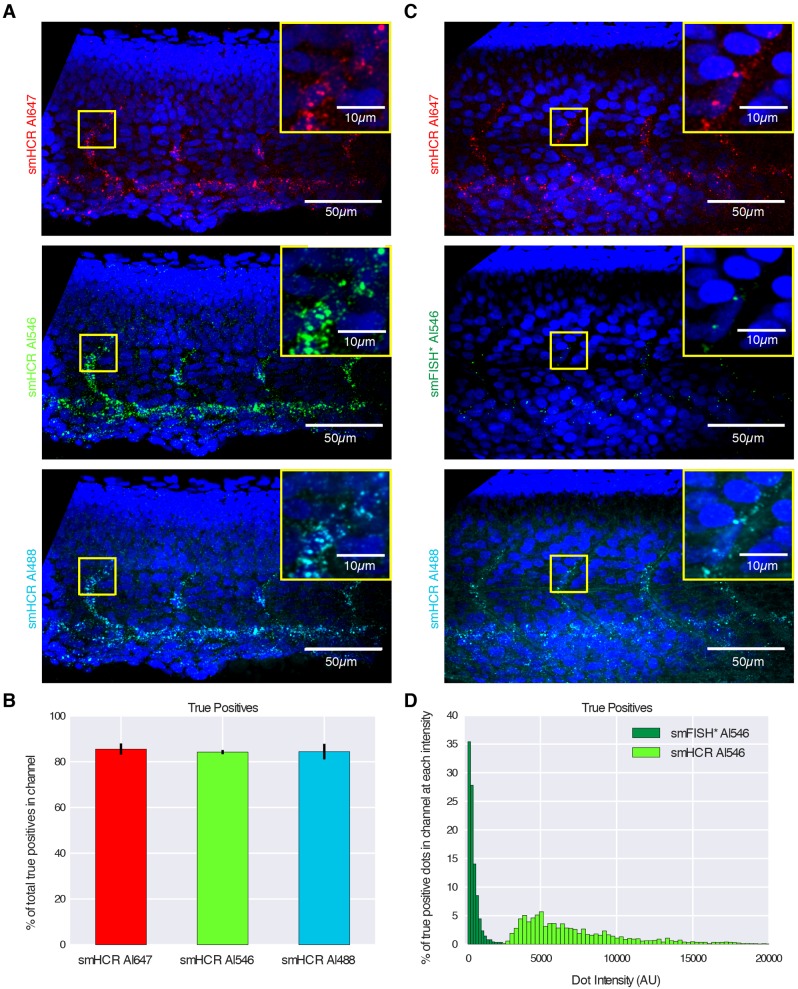
**Imaging single mRNAs within whole-mount zebrafish embryos using smHCR.** (A) Dot colocalization in three channels (DAPI in blue): smHCR (Alexa Fluor 647), smHCR (Alexa Fluor 546), smHCR (Alexa Fluor 488). (B) True positive rates for each channel in A (median±median absolute deviation, *N*=6 embryos). (C) Comparison of smHCR and smFISH* via dot colocalization in three channels: smHCR (Alexa Fluor 647), smFISH* (Alexa Fluor 546), smHCR (Alexa Fluor 488). Channel pairs between A and C are shown with the same contrast; Alexa Fluor 546 images illustrate the difference in intensity between amplified smHCR dots and unamplified smFISH* dots. (D) True positive dot intensities for smHCR (Alexa Fluor 546; *N*=6 embryos) and smFISH* (Alexa Fluor 546; *N*=3 embryos). Target mRNA: *kdrl* (expressed in the endothelial cells of blood vessels). Microscopy: spinning disk confocal. Probe sets: 39 probes per set, each addressing a 30 nt target subsequence. Embryos fixed: 27 hpf. See Figs S6-S8 for additional data.

To examine the performance of smHCR within the more challenging imaging setting of an intact vertebrate embryo, we repeated the three-channel colocalization study in a whole-mount zebrafish embryo with confocal imaging. The target mRNA, *kdrl* (a medium-copy target expressed in the endothelial cells of blood vessels), is detected using three smHCR probe sets of 39 probes each, with probes alternating between the three sets along the target mRNA (Table S3). For the three smHCR channels, we observe true positive rates of 86%, 84% and 86% (Fig. 2B), and false positive rates of 36%, 21% and 31% (Fig. S6). To compare the properties of smHCR and smFISH in zebrafish embryos, we disabled HCR amplification in the Alexa Fluor 546 channel by introducing only the first HCR hairpin species, enabling only one hairpin carrying one fluorophore to bind to each probe (smFISH* in Fig. 2C; compare the dots in the middle panels of A and C). Comparing the signal intensities of smHCR and smFISH*, we find that the ratio of median dot intensities for smHCR and smFISH* is approximately 15 (Fig. 2D). While the autofluorescence in zebrafish embryos is low enough that unamplified smFISH remains viable (Oka and Sato, 2015; Stapel et al., 2016; Figs S6-S8), automated signal detection is facilitated by the greater signal to background ratio of smHCR.

**Fig. 3.**
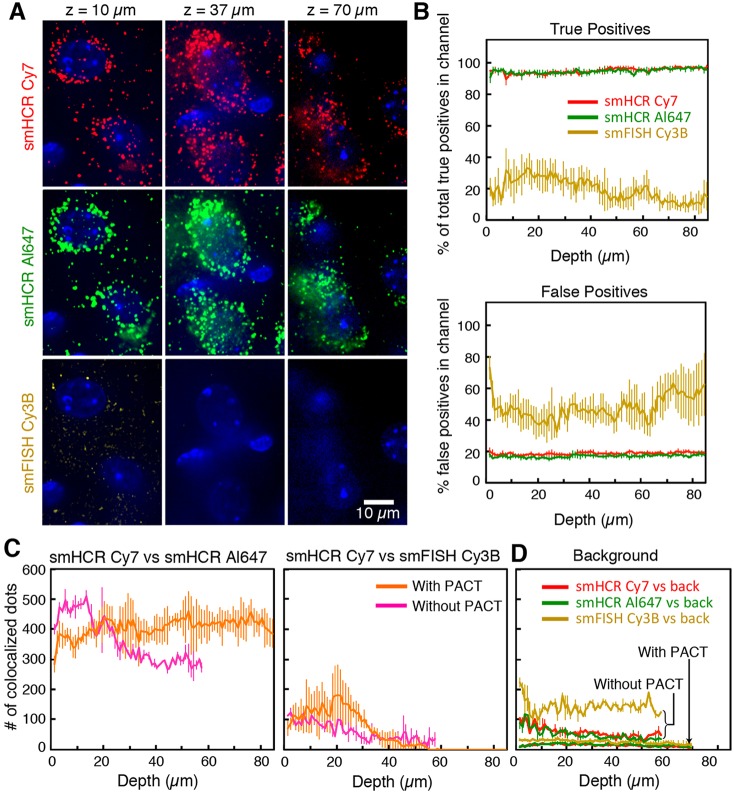
**Imaging single mRNAs in adult mouse brain sections using smHCR and PACT.** (A) Dot colocalization in three channels at three depths (DAPI in blue): smHCR (Cy7), smHCR (Alexa Fluor 647), smFISH (Cy3B). Images are displayed with the same contrast within each row. (B) True positive and false positive rates as a function of depth (median±median absolute deviation, *N*=8 sections). (C) Effect of PACT clearing on the absolute number of colocalized dots for pairs of channels as a function of depth within a 110×110×1 µm voxel (median±median absolute deviation, *N*=8 sections with PACT, *N=*3 sections without PACT). (D) Characterization of background with and without PACT via colocalization of dots in any of three channels with dots due solely to autofluorescence in a fourth channel (excitation at 589 nm) (median±median absolute deviation, *N*=6 sections with PACT, *N=*3 sections without PACT). Target: *Pgk1*. Microscopy: spinning disk confocal. Probe sets: 22 or 23 probes per set, each addressing a 20 nt target subsequence. See Fig. S9 for additional data.

In the higher background of adult mouse brain sections, smHCR signal amplification becomes essential for robust detection of individual transcripts and is also important when mapping bulk expression in cleared tissue (Sylwestrak et al., 2016). To minimize autofluorescence and light scattering, PACT clearing turns tissues optically transparent and macromolecule-permeable by removing lipids and replacing them with a porous hydrogel, while immersion in RIMS matches the refractive index throughout the sample (Treweek et al., 2015; Yang et al., 2014). We hypothesized that PACT and RIMS combined with smHCR should enable reliable single-molecule imaging at depth within brain samples. As a first test, we used confocal microscopy to image PACT-cleared brain slices at depths up to 84 µm (Fig. 3), performing a three-channel colocalization study using two smHCR probe sets and one smFISH probe set (Table S4). Using smFISH, few dots are visible at a depth of 10 µm, and no signal is evident at a depth of 37 µm (Fig. 3A, bottom). This situation contrasts with the two smHCR channels, where dots remain bright at a depth of 70 µm (Fig. 3A, middle and top). For each of the two smHCR channels, the true positive rate is approximately >90% (Fig. 3B, top) and the false positive rate is approximately 20% (Fig. 3B, bottom) across the full range of depths. For the smFISH channel, the true positive rate is dramatically lower and the false positive rate is dramatically higher at all depths (Fig. 3B).

To further examine the role of tissue clearing in smRNA detection at depth, we calculated the absolute number of colocalized dots per imaging voxel for pairs of channels with and without PACT (Fig. 3C). Owing to the high level and ubiquitous nature of *Pgk1* expression, two samples when imaged in the same relative locations (layer I-layer II/III of parietal cortex) should show similar transcript numbers per unit volume. With PACT, the two smHCR channels show no measurable decline in colocalized dot count as a function of depth, but without PACT, the dot count decreases at depths beyond ∼15 µm (Fig. 3C left). PACT also significantly reduces the background dot count resulting from autofluorescence (Fig. 3D). Comparisons between smHCR and smFISH emphasize the lack of signal using smFISH (Fig. 3C, right).

**Fig. 4.**
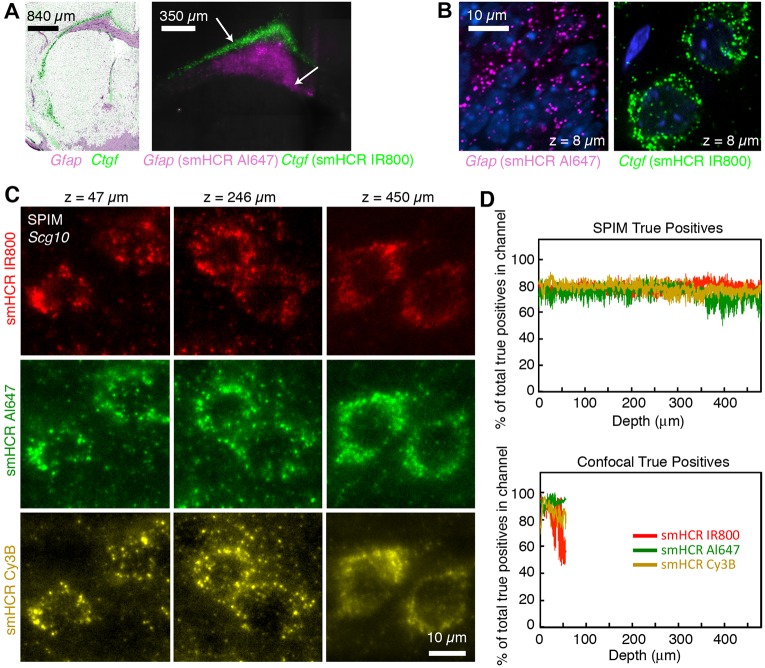
**Imaging single mRNAs in thick adult mouse brain sections using smHCR, PACT and SPIM.** (A) *Ctgf* and *Gfap* expression based on: reference Allen Brain Atlas (ABA) composite (*Ctgf*-RP_040407_02_H07-coronal slice #16 and *Gfap*-RP_Baylor_253913 coronal slice #16, sections were selected and overlaid based on the ventricle outline) (left panel) and two-channel smHCR with PACT and SPIM (right panel). Images were generated by stitching together tiled fields of view (1.2×1.2 mm) acquired in SPIM. The SPIM image shows a maximum intensity projection of a 250 µm stack of images. Consistent with the ABA-based overlay, the SPIM images show *Ctgf* highly expressed in the deepest cortical layer and *Gfap* expressed in the white matter astrocytes. (B) High-magnification confocal images at two locations within the same sample (approximate locations denoted by arrows in A). DAPI in blue. (C) *Scg10* mRNA imaged in three channels at three depths using smHCR (IR800, Alexa Fluor 647, Cy3B), PACT and SPIM. (D) True positive rates as a function of depth (median±median absolute deviation, *N*=3 sections from different brains) using SPIM or confocal imaging of *Scg10*. Probe sets: 20 probes per set, each addressing a 20 nt target subsequence. See Fig. S10 and Movie 2 for additional data.

Although confocal microscopy rejects out-of-focus background, image acquisition is slow, out-of-plane excitation can photobleach the sample and the imaging depth is limited compared with SPIM. SPIM (Huisken et al., 2004; Dodt et al., 2007; Keller et al., 2010; Tomer et al., 2014; Baumgart and Kubitscheck, 2012) offers a fast alternative (∼100 times faster than confocal microscopy) that rejects out-of-focus noise by illuminating and capturing images only from a thin selective plane, typically on the order of 1-10 µm. As smFISH signal is undetectable with SPIM, if SPIM, PACT and smHCR are compatible, it would become feasible to efficiently perform phenotypical studies with single-molecule resolution while preserving the natural long-range architecture of thick samples. To examine the performance of SPIM, PACT and smHCR, we first mapped the expression patterns for two mRNAs (*Ctgf* and *Gfap*) in 250 μm brain slices, recapitulating the large-scale reference patterns in the Allen Brain Atlas (ABA) (Fig. 4A and Movie 1), but now with single-molecule resolution (Fig. 4B). To further characterize SPIM performance, we mapped single *Scg10* mRNAs (a medium to high copy number target) at depths up to 0.5 mm in PACT-cleared brain slices (Fig. 4C and Movie 2). Examining true positive (Fig. 4D) and false positive (Fig. S10) rates for three smHCR channels reveals that SPIM extends the sensitivity and selectivity achieved with confocal microscopy to significantly greater depths (see Figs S11 and S12 for an illustration of image analysis and dot classification for smHCR/PACT/SPIM data in thick samples). Additional studies mapping a high-copy transgenic mRNA in 1 mm brain slices from Thy1-EYFP mice revealed strong and selective HCR signal at depth (Figs S13-S16 and Movie 3), although in this case the expression level of the target was too high to resolve individual dots. Notably though, as PACT-cleared tissue retains endogenous YFP fluorescence, we were able to directly test the selectivity of HCR signal without the need for parallel antibody staining; we observe a one-to-one correspondence between cells labeled by YFP protein fluorescence and cells expressing YFP mRNA by smHCR.

In conclusion, we have shown that smHCR provides a robust method to map single mRNAs of varying abundance in diverse samples. In combination with PACT and SPIM, smHCR enables efficient mapping of single transcripts in thick brain slices, allowing the spatial architecture of the tissue to be preserved. Noting that whole bodies and a wide range of tissues, including human, have been successfully cleared (Treweek et al., 2015; Yang et al., 2014), we expect the combination of smHCR, PACT and SPIM to enable molecular profiling of a wide variety of samples with single-cell and, if desired, single-transcript resolution while preserving geometry and connectivity information. As smHCR is compatible with sequential hybridization methods that we have previously developed (Lubeck et al., 2014; Lubeck and Cai, 2012), it should be possible to perform highly multiplexed studies within thick autofluorescent samples, mapping single mRNAs at depth.

## MATERIALS AND METHODS

### Sample preparation

Whole-mount zebrafish embryos were prepared using a protocol adapted from Choi et al. (2014). Mouse CNS-derived CAD cells and adult mouse brain sections up to 1 mm thick were prepared using standard techniques as described in supplementary Materials and Methods.

### *In situ* hybridization

smFISH and smHCR probe preparation, and specific hybridization conditions for cultured cells, zebrafish and mouse brain sections are described in supplementary Materials and Methods.

### Passive CLARITY technique

Paraformaldehyde-fixed mouse brain slices were PACT cleared before imaging following a previously reported protocol (Yang et al., 2014; Treweek et al., 2015) as detailed in supplementary Materials and Methods.

### Imaging

Epifluorescence microscopy, spinning disk confocal microscopy or SPIM was carried out on cultured cells, whole-mount zebrafish and adult mouse sections as described in supplementary Materials and Methods.

### Image processing and analysis

All image analysis of cultured cells, whole-mount zebrafish and adult mouse sections was performed on three-dimensional image stacks in MATLAB as detailed in supplementary Materials and Methods.
